# Parathyroid hormone and premature thymus ageing in patients with chronic kidney disease

**DOI:** 10.1038/s41598-018-37511-9

**Published:** 2019-01-28

**Authors:** Kenichiro Iio, Daijiro Kabata, Rei Iio, Yosuke Imai, Masaki Hatanaka, Hiroki Omori, Yoshihiko Hoshida, Yukihiko Saeki, Ayumi Shintani, Takayuki Hamano, Yoshitaka Isaka, Yutaka Ando

**Affiliations:** 1 Department of Nephrology, National Hospital Organization Osaka Minami Medical Center, Kawachinagano, Japan; 2 Department of Medical Statistics, Osaka City University Graduate School of Medicine, Osaka, Japan; 3 Department of Kidney Disease and Hypertension, Osaka General Medical Center, Osaka, Japan; 4 Department of Pathology, National Hospital Organization Osaka Minami Medical Center, Kawachinagano, Japan; 5 Department of Clinical Research, National Hospital Organization Osaka Minami Medical Center, Kawachinagano, Japan; 6 Department of Inter-Organ Communication Research in Kidney Disease, Osaka University Graduate School of Medicine, Suita, Japan; 7 Department of Nephrology, Osaka University Graduate School of Medicine, Suita, Japan

## Abstract

Premature immune ageing, including thymic atrophy, is observed in patients with chronic kidney disease (CKD). Parathyroid hormone (PTH) and fibroblast growth factor 23 (FGF23), which are mineral and bone disorder (MBD)-related factors, affect immune cells and possibly cause thymic atrophy. We examined the cross-sectional association between thymic atrophy, evaluated as the number of CD3^+^CD4^+^CD45RA^+^CD31^+^ cells [recent thymic emigrants (RTE)/μL], and MBD-related factors [(serum PTH, FGF23, and alkaline phosphatase (ALP) level] in 125 patients with non-dialysis dependent CKD. Median estimated glomerular filtration rate (eGFR) was 17 mL/min/1.73 m^2^. Older age (*r* = −0.46), male sex (*r* = −0.34), lower eGFR (*r* = 0.27), lower serum-corrected calcium (*r* = 0.27), higher PTH (*r* = −0.36), and higher ALP level (*r* = −0.20) were identified as determinants of lower number of RTE. In contrast, serum concentrations of FGF23 and phosphorus were not correlated with RTE. Multivariate non-linear regression analysis indicated a negative association between serum PTH and log-transformed RTE (P = 0.030, P for non-linearity = 0.124). However, the serum levels of FGF23 and ALP were not associated with RTE. In patients with CKD, serum PTH concentrations were related to thymic atrophy which contributes to immune abnormality.

## Introduction

Chronic kidney disease (CKD) promotes premature immune ageing, including thymic atrophy, which might be associated with high mortality due to infection or cardiovascular disease (CVD)^1–3^. The thymus generates T cells, and upon atrophy, the supply of naïve T cells and the diversity of the cell repertoire diminish. The loss of such diversity results in susceptibility to infection due to insufficient responses to new pathogens. Patients with DiGeorge syndrome, in which the thymus is congenitally hypoplastic, become infected easily due to T-cell deficiency^4^. When the number of naïve T cells decreases and the diversity of the repertoire is lost, effector T-cell population increases. This condition is related to chronic inflammation and arteriosclerosis^5^.

However, the specific CKD pathology that is associated with thymic atrophy remains unknown. CKD is a disease in which the function of the kidneys gradually decreases. In patients with CKD, mineral and bone disease (MBD) can cause abnormal serum concentration of calcium, phosphorus, parathyroid hormone (PTH), fibroblast growth factor 23 (FGF23), and alkaline phosphatase (ALP), resulting in bone-related abnormalities and vascular calcification. This complication is associated with increased mortality by CVD or infectious disease^6–14^.

In recent years, MBD has been found to act on organs other than bone and vascular calcification. Cachexia is mediated by the PTH receptor^15^, and left ventricular hypertrophy is mediated by FGF receptor 4 by the action of FGF23^16^ through direct organ involvement. Additionally, PTH and FGF23 act on immune cells^17,18^ and it exhibits thymic atrophy in klotho-hypomorphic mice and FGF23 knockout mice^19,20^. These facts indicate that MBD might likewise affect the thymus in humans.

Based on this, we hypothesised that MBD is involved in thymic atrophy in human. We examined this hypothesis by investigating the relationship between thymic function and MBD markers (serum PTH, FGF23, and ALP) in patients with non-dialysis dependent CKD.

## Results

### Clinical characteristics

Table 1 shows the clinical characteristics of the 125 patients enrolled in this study, as well as the classification according to eGFR. Twelve (10%), 57 (46%), and 56 (45%) patients exhibited an eGFR (mL/min/1.73 m^2^) ≥60 (CKD stage 1 or 2), 15–60 (CKD stage 3 or 4), and <15 (CKD stage 5), respectively. The median (interquartile range) age was 71 (61, 77) years, and 63% of the participants were men. Pre-existing CVD was evident in 21% of patients. The aetiologies of kidney disease comprised diabetic nephropathy (n = 40, 32%), glomerulonephritis (n = 46, 37%), nephrosclerosis (n = 22, 18%), and others (n = 17, 14%). Glomerulonephritis was frequent (83%) in the group with eGFR ≥60 mL/min/1.73 m^2^. A phosphate binder was used only when eGFR was <15 mL/min/1.73 m^2^ (43% of patients). Active vitamin D was used when eGFR was 15–60 mL/min/1.73 m^2^ (9% of patients) or <15 mL/min/1.73 m^2^ (59% of patients). The serum-corrected calcium level did not change even when eGFR decreased. The median serum phosphorus level was 5.1 mg/dL with eGFR <15 mL/min/1.73 m^2^. Serum levels of both PTH and FGF23 increased as eGFR decreased, but serum ALP level did not change despite the decrease in eGFR.

**Table 1:** Characteristics of the study population.

Variable	All n = 125		eGFR (mL/min/1.73 m^2^)						Missing (%)
			eGFR ≥ 60 n = 12		15 ≤ eGFR < 60 n = 57		eGFR < 15 n = 56		
Age (years)	71	[61, 77]^¶^	45	[35, 60]	73	[63, 77]	70	[64, 77]	0
Male, n (%)	79	(63)	3	(25)	41	(72)	35	(63)	0
CVD, n (%)	26	(21)	1	(8)	15	(26)	10	(18)	0
Current smoking, n (%)	24	(20)	3	(25)	12	(21)	9	(17)	4.0
Hypertension, n (%)	108	(86)	2	(17)	53	(93)	53	(95)	0
Diabetes, n (%)	53	(42)	3	(25)	25	(44)	25	(45)	0
**Primary renal disease**									
Diabetic nephropathy, n (%)	40	(32)	2	(17)	18	(32)	20	(36)	0
Glomerulonephritis, n (%)	46	(37)	10	(83)	20	(35)	16	(29)	0
Nephrosclerosis, n (%)	22	(18)	0	(0)	9	(16)	13	(23)	0
Others, n (%)	17	(14)	0	(0)	10	(18)	7	(13)	0
BMI (kg/m^2^)	23.0	[20.0, 25.5]	20.9	[18.3, 23.9]	24.1	[21.4, 25.5]	22.7	[19.8, 25.6]	2.4
Prior immunosuppression, n (%)	9	(7)	0	(0)	6	(11)	3	(5)	0
Phosphate binder, n (%)	24	(19)	0	(0)	0	(0)	24	(43)	0
Active vitamin D supplementation, n (%)	38	(30)	0	(0)	5	(9)	33	(59)	0
eGFR (mL/min/1.73 m^2^)	17	[6, 37]	89	[68, 100]	32	[23, 40]	5	[4, 9]	0
Haemoglobin (g/dL)	11.0	[10.1, 13.0]	13.5	[12.7, 14.1]	12.6	[11.1, 14.3]	10.1	[9.3, 10.7]	0
Serum corrected calcium (mg/dL)	9.3	[8.9, 9.6]	9.5	[9.4, 9.7]	9.3	[9.0, 9.6]	9.3	[8.9, 9.6]	0.8
Serum phosphorus (mg/dL)	3.9	[3.3, 5.0]	3.8	[3.3, 3.9]	3.4	[3.1, 3.8]	5.1	[4.6, 6.0]	0.8
Serum FGF23 (pg/mL)	135	[76, 465]	41	[32, 62]	88	[58, 135]	669	[250, 3396]	4.0
Serum intact PTH (pg/mL)	80	[45, 192]	38	[35, 42]	56	[41, 84]	199	[108, 279]	0
Serum ALP (IU/L)	213	[172, 283]	183	[156, 205]	227	[167, 291]	226	[181, 295]	0
Serum albumin (g/dL)	3.8	[3.3, 4.0]	4.1	[3.6, 4.3]	4.0	[3.5, 4.1]	3.6	[3.1, 3.9]	0
Serum CRP (mg/dL)	0.09	[0.03, 0.15]	0.08	[0.02, 0.10]	0.07	[0.04, 0.14]	0.10	[0.03, 0.19]	0
Urinary protein (g/gCr)	2.09	[0.75, 4.00]	0.75	[0.24, 1.23]	1.26	[0.27, 3.21]	3.21	[1.88, 5.09]	0

Data are presented as numbers (%) or ^¶^median [interquartile range]. CVD, cardiovascular disease; BMI, body mass index; eGFR, estimated glomerular filtration rate; FGF23, fibroblast growth factor 23; PTH, parathyroid hormone; ALP, alkaline phosphatase; CRP, C reactive protein.

### Associations between thymic function and clinical and biochemical values

Table 2 shows associations between thymic function and clinical variables. Age and male sex were negatively associated with CD3^+^CD4^+^CD45RA^+^CD31^+^ cells [Recent thymic emigrants (RTEs)/μL] (RTE) and the proportions of CD45RA^+^CD31^+^ cells in the CD3^+^CD4^+^ cell subset (RTE%), as previously reported (age-RTE: *r* = −0.46, P < 0.001; age-RTE%: *r* = −0.44, P < 0.001; male-RTE: *r* = −0.34, P < 0.001; male-RTE%: *r* = −0.40, P < 0.001)^21^. Thymic function is reportedly altered in patients on dialysis^1^. Therefore, we examined correlations between thymic function and eGFR and MBD-related factors and found that eGFR was correlated positively with RTE (*r* = 0.27, P = 0.003). Furthermore, among the variables associated with MBD and thymic atrophy, serum level of PTH and corrected calcium correlated negatively and positively, respectively, with both RTE and RTE% (PTH-RTE: *r* = −0.36, P < 0.001; PTH-RTE%: *r* = −0.21, P = 0.019; Calcium-RTE: *r* = 0.27, P = 0.002; Calcium-RTE%: *r* = 0.18, P = 0.045), whereas serum ALP level was correlated negatively with RTE (*r* = −0.20, P = 0.023). Serum level of FGF23 and phosphorus correlated with neither RTE nor RTE%. That is, thymic function correlated with eGFR as well as the serum concentration of PTH, ALP, and corrected calcium, which are factors related to secondary hyperparathyroidism, in addition to age and male sex, but not with the serum concentration of FGF23 or phosphorus, which are associated with phosphorus metabolism.

**Table 2:** Correlation between RTE or RTE% and characteristics of participants.

	RTE		RTE%	
	*r*	P	*r*	P
Age	−0.46	<0.001	−0.44	<0.001
Male	−0.34	<0.001	−0.40	<0.001
eGFR	0.27	0.003	0.13	0.146
Serum PTH	−0.36	<0.001	−0.21	0.019
Serum FGF23	−0.099	0.281	0.022	0.812
Serum ALP	−0.20	0.023	−0.14	0.112
Serum-corrected calcium	0.27	0.002	0.18	0.045
Serum phosphorus	0.017	0.848	0.15	0.097

RTE, recent thymic emigrants; RTE%, the proportion of recent thymic emigrants among CD4^+^ T cells; eGFR, estimated glomerular filtration rate; PTH, parathyroid hormone; FGF23, fibroblast growth factor 23; ALP, alkaline phosphatase.

### Determinants of LnRTE and RTE%

**Figure 1:**
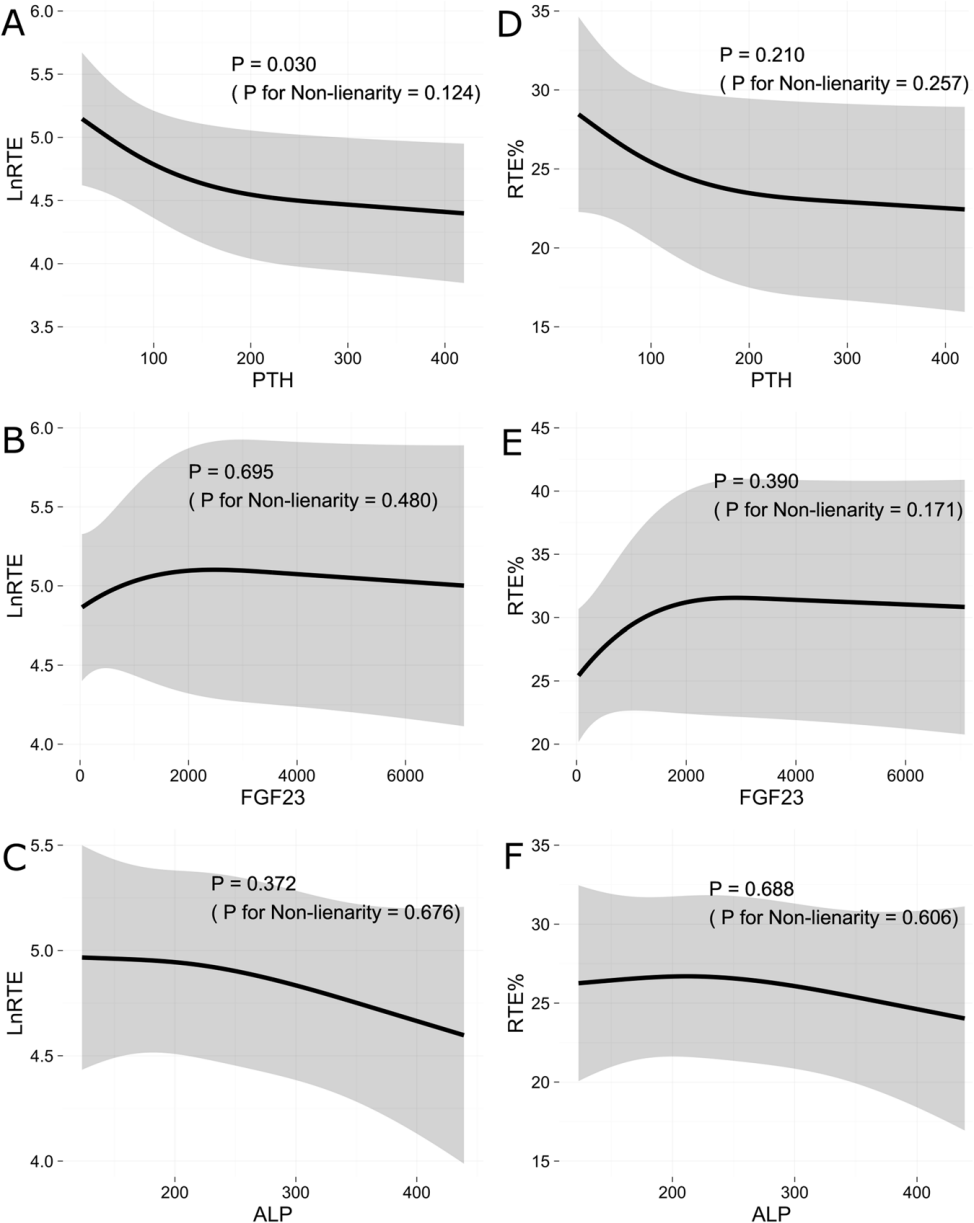
Association between LnRTE (log-transformed RTE, RTE = the number of CD3^+^CD4^+^CD45RA^+^CD31^+^ cells/µL) or RTE% [proportion of RTEs (CD45RA^+^CD31^+^ cells in CD3^+^CD4^+^ cell populations)] and mineral and bone disorder (MBD)-related factors. Association between LnRTE and MBD-related factors [serum parathyroid hormone (PTH; **A**), fibroblast growth factor 23 (FGF23; **B**), and alkaline phosphatase (ALP; **C**)] in patients with non-dialysis-dependent chronic kidney disease analysed by multivariate non-linear regression analysis. Association between RTE% and MBD-related factors [serum PTH (**D**), FGF23 (**E**), and ALP (**F**)] in patients with non-dialysis-dependent chronic kidney disease analysed by multivariate non-linear regression analysis. The model was adjusted for age, sex, eGFR, diabetes, serum-corrected calcium, serum phosphorus, active vitamin D supplementation, and use of phosphate binder.

We next performed multivariate, non-linear regression analyses to detect variables explaining LnRTE (logarithmically transformed RTE to achieve normality of the residuals). Figure 1(A) shows that LnRTE was significantly correlated with serum PTH level (P = 0.030, P for non-linearity = 0.124) after adjustment for age, male sex, eGFR, diabetes, serum-corrected calcium, serum phosphorus, active vitamin D supplementation, and use of phosphate binders. Neither serum level of FGF23 nor ALP was associated with LnRTE (P = 0.695 or 0.372, P for non-linearity = 0.480 or 0.676, respectively) in Fig. 1(B,C).

Figure 1(D–F) shows the results of a multivariate non-linear regression analysis of variables that explain RTE%. Although not significant, RTE% decreased when serum PTH levels were high, a tendency that was similar to that shown in Fig. 1A (P = 0.210, P for non-linearity = 0.257). Serum levels of FGF23 and ALP were not associated with RTE% (P = 0.390 or 0.688; P for non-linearity = 0.171 or 0.606, respectively). These results indicated that serum PTH level was associated with thymic atrophy in humans, whereas serum FGF23 level was not.

Then, we examined whether active vitamin D supplementation was related to RTE or RTE% as active vitamin D might affect both thymic function and serum PTH level. As shown in Supplemental Table. S1, the relation between RTE or RTE% and active vitamin D supplementation adjusted for age, male sex, eGFR, diabetes, serum-corrected calcium, serum phosphorus, and use of phosphate binder by non-linear multiple regression analysis was examined. There was no association between active vitamin D supplementation and thymic function.

## Discussion

This study showed that thymic atrophy was associated with higher PTH level, but not with FGF23 or ALP level, after adjustment for eGFR, active vitamin D supplementation, and other variables. One mechanism that could explain the association between serum PTH level and thymic atrophy is the effect of PTH activity on organs because PTH receptors are found throughout the body, and thus this hormone can act on organs as well as bones^22^. For example, the effect of PTH on fat and muscle cells causes cachexia in patients with CKD^15^. PTH receptors are also expressed on T cells, and mediate the secretion of Wnt10b, which plays an important role in the osteogenic action of PTH^18^. The action of PTH in bone is mediated through cAMP/PKA and Wnt-independent activation of β-catenin/T-cell factor (TCF) signalling^18,23^. Continuous infusion of PTH also acts on GαS on naïve T cells and promote their differentiation to Th17^24^. As a result, the output from the thymus might be depleted through the loss of naïve T cells. In addition, β-catenin/TCF signalling in T cells is involved in differentiation regulation in the thymus and peripheral tissue^25^. Increased Wnt signalling by PTH in thymic epithelial cells might lead to decreased thymic function^26^. An additional mechanism associated with thymic atrophy caused by PTH is the effect of haematopoietic stem cells. Lymphoid progenitor cells differentiate from haematopoietic stem cells and become thymocytes upon reaching the thymus. Therefore, a decrease in the number of haematopoietic stem cells results in downstream thymic atrophy^27^. Thymic atrophy might also occur via a reduction in the number of haematopoietic stem cells caused by PTH, because patients with CKD have fewer CD34-positive cells, including haematopoietic stem cells than healthy individuals^28^, and serum PTH is associated with a decreased number of CD34-positive cells^29^. Although intermittent PTH (1–34) administration expands haematopoietic stem cells^30–32^, persistent PTH elevation in CKD patients might decrease haematopoietic stem cells due to exhaustion in a manner similar to the sulfonylurea-induced exhaustion of insulin secretion that occurs during diabetes treatment^33^. To understand the role of PTH in thymic atrophy, thymic epithelial cell-specific knockout of PTH receptors in mice may be performed.

Thymic atrophy was not found to be associated with serum FGF23 level in this study, whereas it has been shown to develop in FGF23 knockout^19^ and klotho-hypomorphic mice^20^. Notably, hyperphosphatemia is also associated with thymic atrophy in mice, because crossing klotho-hypomorphic mice with *Npt2a* knockout mice improves thymic atrophy^34^. Although neither serum level of FGF23 nor phosphorus were found to be associated with thymic atrophy in the present study, there is a possibility that it was not related to phosphorus metabolism because of the differences between humans and mice.

We measured RTEs by flow cytometry^35^ to evaluate thymic atrophy, as this is an established method that is used to assess thymic function^21,35,36^. The number of naïve T cells derived from the thymus before an antigen encounter are usually maintained by the peripheral division. However, RTEs are naïve T cells that divide at a lower frequency, and these reflect the production of T cells by the thymus^35^. Thymic atrophy can also be evaluated by measuring T-cell receptor excision circles. This method closely correlates with measuring RTEs by flow cytometry, but it includes PBMC separation and PCR^35^, and its application is subject to considerable variation^35^. The size of the thymus can be measured using chest CT, but this is not quantitative and depends on observer subjectivity^37^. Therefore, quantitative flow cytometry is the most practical method for evaluating thymic atrophy.

PTH was associated with RTE in multivariate analysis in this study. However, RTE% is not significantly associated with PTH in multivariate analysis although RTE% correlated with PTH in univariate analysis and tended to be associated with PTH in multivariate analysis. RTE has a higher correlation coefficient with T-cell receptor excitation circles, which is another indicator of RTEs, than RTE%^38^. This suggests that RTE is thought to reflect more thymic atrophy than RTE%.

In this study, it was demonstrated that thymic atrophy in CKD may be promoted by PTH. Because lower serum calcium and higher ALP are also correlated with thymic atrophy, it can be said that secondary hyperparathyroidism is associated with thymic atrophy. Thymic atrophy reduces repertoire diversity and increases susceptibility to infection^1,2^. In addition, one study has found an association between thymic atrophy and CVD^39^. In another study, anti-thymocyte globulin increases the incidence of CVD when administered to patients receiving renal transplantation^40^. Since atherosclerosis comprises vascular wall inflammation that is affected by the immune system, thymic atrophy in association with immune ageing might cause CVD. Serum PTH level is associated with the onset of CVD, infectious disease, and mortality in an epidemiological study^7,10,12,14^. For these reasons, PTH may be involved in the pathogenesis of these complications by inducing immune abnormality through thymic atrophy in addition to regulating bone metabolism.

This study had several limitations. The observational design cannot determine causal relationships and variables such as 25(OH)D or 1,25(OH)_2_D that was not evaluated might have been confounders. However, multivariate regression analysis did not associate oral active vitamin D supplementation with thymic function (Supplemental Table. S1). Because we evaluated relatively few patients, multiple regression analyses could not include a sufficient number of variables; thus, some factors could not be corrected. Although more related factors might be uncovered by increasing the number of patients, serum PTH is considered to be very closely associated with the thymic function.

In conclusion, we showed that serum PTH concentrations are associated with thymic atrophy. Although this is a cross-sectional study, PTH may cause thymus atrophy and contribute to immune abnormality, which may also partially explain the increased mortality due to elevated PTH level. Further investigation is required to explain the relationship between PTH and thymic atrophy.

## Methods

### Patients

The Ethics Committee at Osaka Minami Medical Center approved this study (25-11, 28-13) and the study was conducted according to the ethical principles of the Declaration of Helsinki. Informed consent was obtained from all the participants. We enrolled 125 patients with non-dialysis dependent CKD who were hospitalised between June 2013 and October 2017. The effect of an acute decline in kidney function and inflammation was avoided by applying the following exclusion criteria: age <20 years old, acute kidney injury, hepatitis B or C virus infection, use of immunosuppressive agents, active infection, and inflammatory diseases such as systemic lupus erythematosus, rheumatoid arthritis, and vasculitis.

### Data collection

Demographic and clinical data at the time of study initiation were collected from the medical records of Osaka Minami Medical Center, and included age, sex, body mass index [BMI; weight (kg)/height (m^2^)], the presence or absence of hypertension and diabetes, haemoglobin, serum levels of calcium, phosphorus, intact PTH, ALP, albumin, C reactive protein, urinary protein, medications (phosphate binder and active vitamin D), prior immunosuppression, and smoking status. Estimated glomerular filtration rate (eGFR) (mL/min/1.73 m^2^) was calculated using the following equation^41^:

$$194\times {\rm{age}}\,{({\rm{y}})}^{-0.287}\times {\rm{serum}}\,{\rm{creatinine}}\,{({\rm{mg}}/{\rm{dL}})}^{-1.094}(\times 0.739\,{\rm{if}}\,{\rm{female}}).$$

Serum calcium levels were corrected based on serum albumin levels as follows: if serum albumin was <4.0 g/dL, then serum calcium (mg/dL) = measured serum calcium (mg/dL) + [4.0 − serum albumin level (g/dL)]^42^. Previous CVD was defined as a history of stroke, coronary artery disease, or peripheral arterial disease. FGF23 was measured using an ELISA kit (Kainos Laboratories Inc, Tokyo, Japan) according to the manufacturer’s protocol.

### Flow cytometry

**Figure 2:**
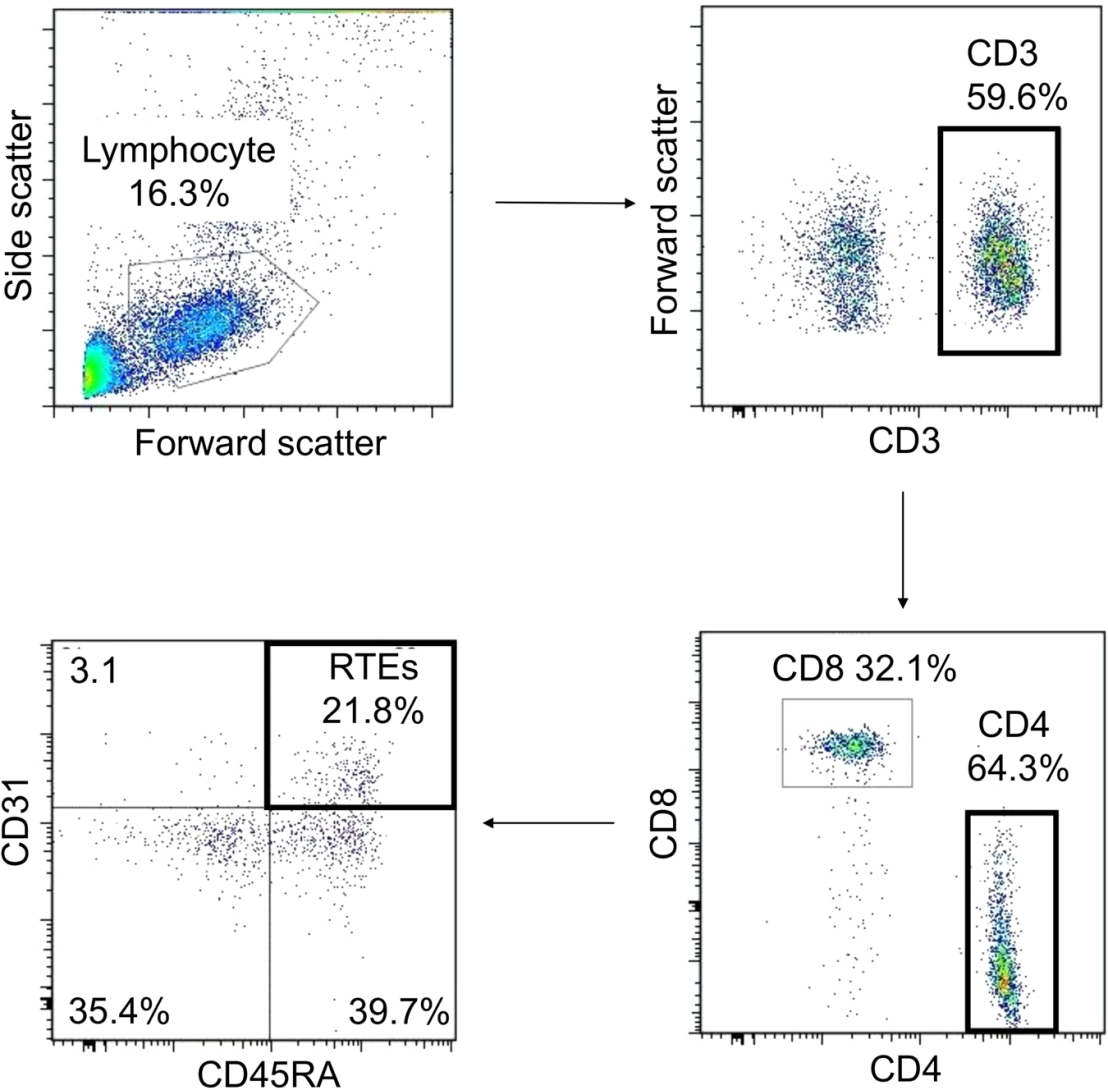
Representative data from flow cytometric detection of CD3^+^CD4^+^CD45RA^+^CD31^+^ cells (recent thymic emigrants, RTEs).

We used PBMCs or peripheral blood to detect T cells by flow cytometry. PBMCs were isolated by density gradient centrifugation using Ficoll-Paque PLUS (GE Healthcare, Uppsala, Sweden) and stored in liquid nitrogen. Serum aliquots from each patient were stored at −20 °C in our biorepository. Cryopreserved PBMCs were thawed and washed twice in RPMI (Wako Pure Chemical Industries, Osaka, Japan) containing 10% foetal calf serum (complete medium). Peripheral blood and PBMCs were stained with the following conjugated antibodies: CD3-PE-Cy7 (mouse IgG1 k, clone UCHT1, Biolegend, Ozyme, Saint-Quentin en Yvelines, France, #300420), CD4-FITC (mouse IgG1 k, clone RPA-T4, Biolegend, #300506), CD8-PerCP-Cy5.5 (mouse IgG1 k, clone RPA-T8, Biolegend, #301032), CD31-PE (mouse IgG1 k, clone WM59, Biolegend, #303105), and CD45RA-ECD (mouse IgG1, clone 2H4LDH11LDB9, Beckman-Coulter, Villepinte, France, #IM2711U). Samples were acquired by using Navios (Beckman), and data were analysed by FlowJo software (Treestar, San Carlos, CA, USA). CD3^+^CD4^+^CD45RA^+^CD31^+^ T cells were defined as RTEs as described in a previous report^35^, and they are expressed as RTE and RTE%. The absolute number of RTE in the peripheral blood was calculated based on the number of peripheral blood lymphocytes at the time of sampling. Figure 2 shows the gating strategy for detecting RTEs.

### Statistical analyses

Data are presented as medians (interquartile ranges). Categorical variables are presented as numbers (%). After classification based on eGFR (<15, 15–60, or ≥60 mL/min/1.73 m^2^), differences in the clinical characteristics of patients were compared using Kruskal-Wallis tests for continuous variables and chi-squared tests for categorical variables. Correlations were assessed by calculating Spearman’s correlation coefficients. To examine the effect of serum PTH level on RTE and RTE%, multivariate non-linear regression analysis that included RTE and RTE% as dependent variables were conducted separately with adjustments for age, sex, eGFR, diabetes, serum-corrected calcium, serum phosphorus, active vitamin D supplementation, and the use of phosphonate binder. To satisfy the assumption of linear regression models that the residuals are normally distributed, RTE was log-transformed. A similar analysis with the above non-linear regression model was performed to assess the impacts of serum FGF23 and ALP level on RTE and RTE% separately. We also performed a multivariable non-linear regression analysis including log-transformed RTE and RTE% separately as a function of active vitamin D supplementation and other covariates described above to assess the association between active vitamin D supplementation and RTE or RTE%. All statistical analyses were used to determine the two-sided significance level at P < 0.05 using R software version 3.3.2 (https://www.r-project.org/foundation/) using the “rms” package^43^.

## Supplementary information

Supplementary Table

## Data Availability

The datasets generated during the current study are available from the corresponding author on reasonable request.
