# Supplementary material for: Parathyroid hormone and premature thymus ageing in patients with chronic kidney disease
Source: Sci Rep. 2019 Jan 28;9:813. doi: 10.1038/s41598-018-37511-9 (PMC6349929; doi:10.1038/s41598-018-37511-9)
Supplement: Supplementary file 1 — Supplementary Table [file 41598_2018_37511_MOESM1_ESM.docx]

Parathyroid hormone and premature thymus ageing in patients with chronic kidney disease

**Kenichiro Iio*^1^, Daijiro Kabata^2^, Rei Iio^3^, Yosuke Imai^1^, Masaki Hatanaka^1^, Hiroki Omori^1^, Yoshihiko Hoshida^4^, Yukihiko Saeki^5^, Ayumi Shintani^2^, Takayuki Hamano^6^, Yoshitaka Isaka^7^, Yutaka Ando^1^**

^1^Department of Nephrology, National Hospital Organization Osaka Minami Medical Center, Kawachinagano, Japan

^2^Department of Medical Statistics, Osaka City University Graduate School of Medicine, Osaka, Japan

^3^Department of Kidney Disease and Hypertension, Osaka General Medical Center, Osaka, Japan

^4^Department of Pathology, National Hospital Organization Osaka Minami Medical Center, Kawachinagano, Japan

^5^Department of Clinical Research, National Hospital Organization Osaka Minami Medical Center, Kawachinagano, Japan

^6^Department of Inter-Organ Communication Research in Kidney Disease, Osaka University Graduate School of Medicine, Suita, Japan

^7^Department of Nephrology, Osaka University Graduate School of Medicine, Suita, Japan

Correspondence to:

Kenichiro Iio

Department of Nephrology, National Hospital Organization Osaka Minami Medical Center,

2-1 Kidohigashimachi Kawachinagano Osaka, Postal Code 586-8521, Japan

Phone: +81-721-53-5761, Fax: +81-721-53-8904

E-mail: [iioken16@outlook.com](mailto:iioken16@outlook.com)

**Supplementary Table. S1. Association between LnRTE or RTE% and active vitamin D supplementation.**

|  | Coefficient | Lower 95% CI | Upper 95% CI | P |
| --- | --- | --- | --- | --- |
| LnRTE | 0.017 | -0.406 | 0.440 | 0.937 |
| RTE% | 0.009 | -0.290 | 0.309 | 0.950 |

LnRTE, log-transformed recent thymic emigrants; RTE%, the proportion of recent thymic emigrants among CD4^+^ T cells; CI, confidence interval
